# *In silico* Driven Redesign of a Clinically Relevant Antibody for the Treatment of GD2 Positive Tumors

**DOI:** 10.1371/journal.pone.0063359

**Published:** 2013-05-16

**Authors:** Mahiuddin Ahmed, Yehuda Goldgur, Jian Hu, Hong-Fen Guo, Nai-Kong V. Cheung

**Affiliations:** 1 Department of Pediatrics, Memorial Sloan-Kettering Cancer Center, New York, New York, United States of America; 2 Structural Biology Program, Memorial Sloan-Kettering Cancer Center, New York, New York, United States of America; Carl-Gustav Carus Technical University-Dresden, Germany

## Abstract

Ganglioside GD2 is a cell surface glycolipid that is highly expressed on cancer cells of neuroectodermal origin, including neuroblastoma, retinoblastoma, melanoma, sarcomas, brain tumors and small cell lung cancer. Monoclonal antibodies (MoAb) that target GD2 have shown clinical efficacy in the treatment of GD2 expressing tumors, and are expected to be the new standard of care for the treatment of pediatric neuroblastoma. In this study, the crystal structure of anti-GD2 murine MoAb 3F8 was solved to 1.65 Å resolution and used as a template for molecular docking simulations of its antigen, the penta-saccharide head group of GD2. Molecular docking revealed a binding motif composed of 12 key interacting amino acid side-chains, involving an extensive network of interactions involving main-chain and side-chain hydrogen bonding, two Pi – CH interactions, and an important charged interaction between Arg95 of the H3 loop with the penultimate sialic acid residue of GD2. Based on *in silico* scanning mutagenesis of the 12 interacting amino acids from the docked 3F8:GD2 model, a single point mutation (Heavy Chain: Gly54Ile) was engineered into a humanized 3F8 (hu3F8) MoAb and found to have a 6–9 fold enhancement in antibody-dependent cell-mediated cytotoxicity of neuroblastoma and melanoma cell lines. With enhanced tumor-killing properties, the re-engineered hu3F8 has the potential be a more effective antibody for the treatment of GD2-positive tumors.

## Introduction

**Figure 1:**
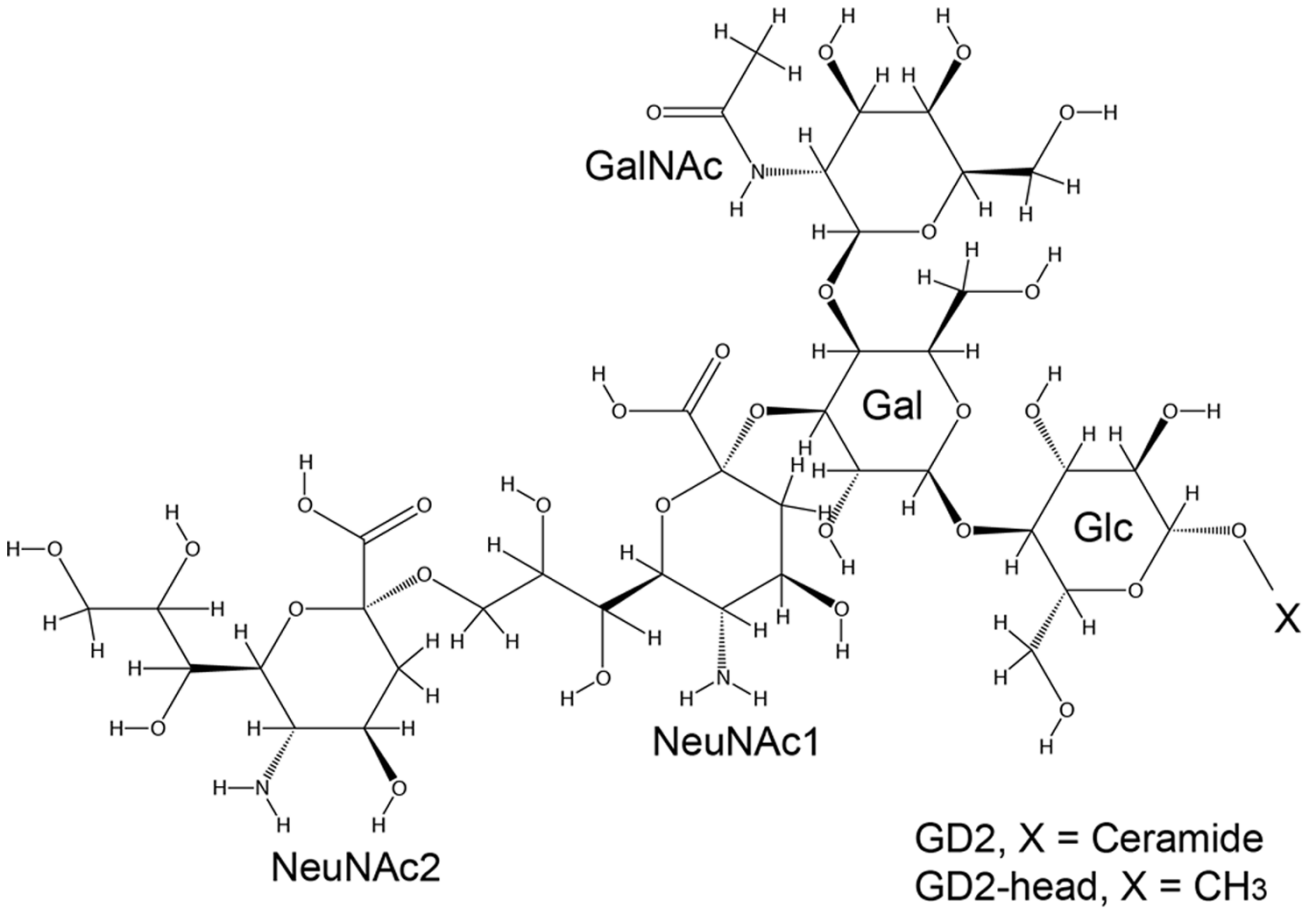
Chemical structure of GD2 ganglioside. Docking studies were performed with GD2-head, where the ceramide moiety was replaced by a methyl group.

Gangliosides are sialic acid containing cell surface glycolipids that have been utilized as targets for cancer immunotherapy [1]. The disialoganglioside GD2, in particular, is a glycolipid antigen that is highly expressed on tumors of both pediatric and adult cancers, including neuroblastoma, retinoblastoma, melanoma, brain tumors, sarcomas and small cell lung cancer [2]. For patients with neuroblastoma, a malignancy accounting for 7% of all childhood cancers and 15% of pediatric cancer deaths, anti-GD2 monoclonal antibody (MoAb) has proven efficacy based on a phase III randomized clinical trial of neuroblastoma patients [3]. Most if not all neuroblastoma tumors express abundant levels of GD2, estimated at 5–10 million molecules/cell with immunosuppressive properties [4]. The structure of GD2 consists of a penta-saccharide head group (containing a glucose Glc, galactose Gal, a branched N-acetylgalactosamine GalNAc, and two sialic acid units NeuNAc1 and NeuNAc2) and a ceramide tail that is embedded in the cell surface membrane (**Figure 1**). GD2 expression in normal tissues is restricted primarily to the central nervous system, with low levels in peripheral nerves and skin melanocytes [5]. The blood brain barrier prevents intravenously administered anti-GD2 MoAbs from entering the central nervous system (CNS), making GD2 an ideal target for neuroectodermal tumors outside of the CNS. Acute toxicities of anti-GD2 MoAb therapy include hypertension, pain, fever and urticaria, although long-term toxicities have been uncommon [2].

3F8 was the first anti-GD2 MoAb to be tested in patients with neuroblastoma [6]. MoAb 3F8 is a murine IgG3 with a moderate affinity for GD2 (K_D_ = 5 nM) [7]. In pre-clinical studies, 3F8 has been shown to have dose-dependent killing of neuroblastoma cells by human complement, and by lymphocytes, cultured monocytes, and granulocytes [2]. 3F8 binds to FcγRII and FcγRIII for neutrophil- and NK-mediated ADCC (antibody-dependent cell-mediated cytotoxicity), respectively, and the CR3 receptor is also important for cytotoxicity [8]. When combined with GM-CSF, 3F8 induced a ∼80% complete response of chemo-resistant NB metastatic to the bone marrow [9], and >60% long term survival among high risk stage 4 children with metastatic neuroblastoma treated in first remission [10]. Murine 3F8 was recently humanized (hu3F8) based on complementarity determining region (CDR) grafting [7], and is currently in Phase I trials (clinical trials.gov NCT01419834 and NCT01662804).

Other anti-GD2 antibodies have also been developed, including murine MoAb ME36.1 and 14G2a, which have lower affinities to GD2 than 3F8 (K_D_ = 19 nM and K_D_ = 77 nM, respectively) [7]. ME36.1 is an IgG2a that binds to GD2 and has modest cross-reactivity to GD3 [11]. 14G2a is also an IgG2a derived by class switching from the IgG3 14.18 MoAb [12]; it was later chimerized to ch14.18 for clinical development [13]. A recent phase III randomized trial showed that ch14.18 when combined with GM-CSF and interleukin-2, was associated with a significantly improved survival in patients with high-risk neuroblastoma [3]. Other forms of antibody based strategies directed at GD2 have also been explored, including immunotoxins [14], immunoliposomes [15], multistep targeting [16] and chimeric immune receptors to retarget T-cells [17].

Given the clinical utility of MoAb against GD2, a better understanding of the structure of anti-GD2 antibodies and how they interact with their antigen is critical in creating new generations of humanized forms with improved therapeutic efficacy. In this investigation, we report the high-resolution crystal structure of murine 3F8 Fab fragment. We then performed computational docking simulations of the GD2 antigen based on the crystal structures of 3F8. Several docking algorithms exist to predict protein:ligand interactions, but few have been utilized to predict protein:oligosaccharide interactions. Protein:oligosaccharides are distinctly different from typical protein:small molecule ligand interactions. Oligosaccharides are often much larger, more flexible, and involve extensive hydrogen bonding and Pi – CH interactions [18]. In addition, the oligosaccharides in ganglioside head groups contain two sialic acid units that are negatively charged and are critical for specificity and affinity of the binding interaction.

Of the numerous computational docking algorithms available [19], only two have been reported in the literature for their potential ability to accurately dock large flexible ligands similar to GD2, namely CDOCKER [20] and GLIDE [21]. For large flexible ligands, CDOCKER was reported to outperform DOCK, FlexX, and GOLD in predicting accurately the docking conformation of ligands with 8 or more rotatable bonds using known co-complexes available in the protein data bank [22]. The ligands tested were, however, much smaller and less flexible than the GD2 head group which has >30 rotatable bonds. GLIDE, on the other hand was reported to outperform AutoDock, GOLD, and FlexX in predicting the binding conformation of oligosaccharides to antibodies as compared to experimentally derived co-crystal structures [18]. However, none of the antigens tested using these algorithms were larger than a tetra-saccharide and none contained charged sialic acid residues. In this investigation, we compared the accuracy of CDOCKER and GLIDE in predicting docked structures of known ligands similar to the pentasaccharide head group of GD2. Based on this analysis, CDOCKER was utilized to create a docked model of GD2 to the crystal structure of 3F8.

Based on the 3F8:GD2 docked model, *in silico* scanning mutagenesis using CHARMm force field methods [23] was then utilized to affinity mature the recently humanized 3F8. To our knowledge, this is the first example of the use of *in silico* scanning mutagenesis to increase antibody affinity to a carbohydrate substrate, and one of the first examples of this technique to affinity mature antibodies in general. Previously, Barberas et al. used homology modeling, docking, and *in silico* mutagenesis to enhance the affinity of human anti-gastrin single chain antibody fragment [24]. Engineered humanized 3F8 MoAbs were then tested for *in vitro* GD2 binding and antibody-dependent cell-mediated cytotoxicity (ADCC) on GD2-positive tumor cell lines.

## Results

### Crystal Structure of 3F8 Fab Fragment

The 3F8 Fab structure was determined by molecular replacement using PDB entry (2AJU) and refined to 1.65 Å resolution. The values for R_cryst_ and R_free_ using the complete diffraction data without sigma cutoff (44,657 reflections) were 17.6 and 22.5% (Table 1). In the high-resolution shell, the R_cryst_ was 22.7% for 3434 reflections (R_free_ 28.9%)in the resolution range 1.71–1.65 Å. The root-mean-square deviation from ideality in bond lengths was 0.024 Å and for bond angles was 2.4°. The electron densities for the entire light chain and the variable region of the heavy chain are of high quality. The heavy chain residues 129–130 were found to be disordered. Disorder in this region has been observed for other Fab structures [25], [26]. Ramachandran analysis showed that 97.1% of the residues fell within the most favored regions.

**Table 1:** Summary of crystallographic analysis.

Resolution (Å)	30–1.65 (1.71–1.65)
Completeness (%)	98.3 (97.4)
Redundancy (fold)	2.5 (2.5)
I/σI	30.3 (3.0)
R_merge_ (%)	3.4 (32.4)
Space group	C2
Cell dimensions (Å)	a = 116.0, b = 57.2, c = 93.7
Refinement	
Reflections working/test	44657/2535 (3434/184)
Residues	428
Solvent	585
R_cryst_/R_free_	17.6/22.5 (22.7/28.9)
R.m.s. deviations	
Bonds (Å)	0.024
Angles (°)	2.357
Ramachandran analysis	
Most favored regions (%)	97.1

Values in parentheses correspond to the high-resolution shell.

Rmerge = Σ|I − <I>|/ΣI, where I = observed intensity and <I> = average intensity obtained from multiple observations of symmetry-related reflections.

The r.m.s. deviations in bond lengths and angles are the respective root-mean-square deviations from ideal values.

**Figure 2:**
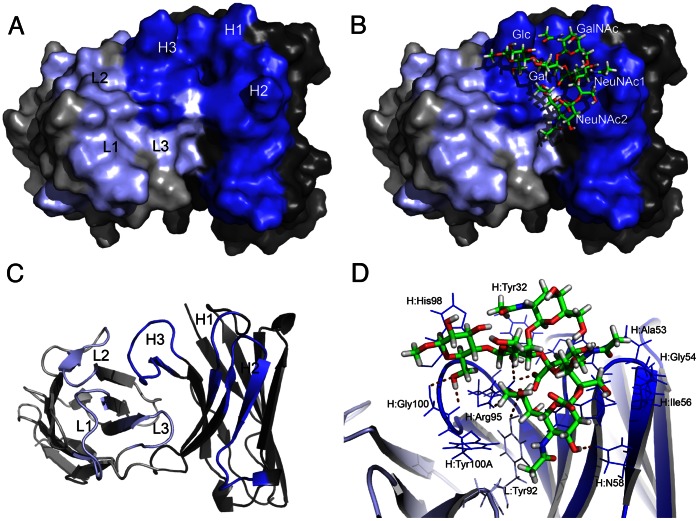
Crystal structure of MoAb 3F8 Fab fragment and docked model with GD2 pentasaccharide head group. A. Space filling model of the antigen binding domain of MoAb 3F8. Heavy Chain CDR loops colored in dark blue; light chain CDR loops colored in light blue. B. Space filling model of 3F8 with docked GD2 pentasaccharide head group. C. Backbone ribbon diagram of 3F8 antigen binding domain. D. Key interacting residues in 3F8:GD2 docked complex.

The 3F8 Fab structure had an immunoglobulin fold common to all Fab structures (Figure 2). The antigen recognition site was formed by the six CDR loops (H1, H2, H3, L1, L2, and L3) (**Figure 2A, 2C**), which had well defined electron densities. The CDR loops formed a binding cavity that was dominated by protrusions from the H3, H1, and H2 loops with a small contribution from the L3 loop. The binding cavity had only two charged residues H:His98 and H:Arg95, which protruded from the H3 loop. The side-chain of H:Arg95 was positioned at the bottom of the binding cavity and was a likely candidate for coordinating the negative charge of a sialic acid group. The rest of the binding cavity was predominantly composed of polar uncharged residues, which could contribute to the extensive hydrogen-bonding network seen with oligosaccharide recognition. Only two hydrophobic residues were found in the vicinity of the binding cavity, namely H:Trp52 and H:Ile56. There were a total of five aromatic residues in the binding cavity: including L:Tyr92, H:Tyr32, H: Trp52, H:His98, and H:Tyr100A. The presence of three tyrosine residues was significant since partial loss of immunoreactivity has been consistently seen during tyrosine iodination of MoAb 3F8 [**27**].

### Computational Docking of GD2 Antigen to 3F8 Crystal Structure

Attempts to form diffractable crystals of 3F8 Fab in complex with soluble derivatives of GD2 were unsuccessful. In the absence of structural data of the co-complex, computational docking algorithms were utilized to model the interaction. To determine which docking algorithm to utilize, a head-to-head comparison between GLIDE and CDOCKER was performed in docking ligands similar to GD2 (sialic acid containing oligosaccharides) using three available test cases of proteins that bind to ganglioside head groups from the protein data bank (PDB code 2HRL: Siglec-7 in complex with GT1b; PDB code 3BWR: Simian virus VP1 in complex with GM1; and PDB code 3HMY: Tetanus toxin HCR/T in complex with GT2). In all three cases, CDOCKER was able to more accurately predict the correct binding conformation of the respective oligosaccharide than GLIDE (**Table 2**). In two of the three test cases (2HRL and 3HMY), CDOCKER was able to predict the binding mode of the respective ligand to a high degree of accuracy (<2 Å RMSD), whereas GLIDE failed in all three cases.

**Table 2:** Comparison of docking algorithms GLIDE versus CDOCKER in predicting known protein:ganglioside complexes.

Protein	PDB code	Native Ligand	Ligand used fordocking	RMSD of topCDOCKER pose (Å)	RMSD of top GLIDE pose (Å)
Tetanus toxin HCR/T	3HMY	GT2	trisaccharide	1.3	9.1
Siglec-7	2HRL	GT1b	pentasaccharide	1.3	8.8
Simian virus VP1	3BWR	GM1	pentasaccharide	6.6	7.9

We therefore chose CDOCKER to dock the GD2 penta-saccharide head group to the antigen-binding pocket of 3F8 (**Figure 2C and 2D**). The top docked structure was then energy minimized using CHARMm force fields. The docking study identified 12 amino acids that directly interacted with the GD2 head group (L:Asp91, L:Tyr92, H:Trp52, H:Ala53, H:Gly54, H:Gly55, H:Ile56, H:Asn58, H:Arg95, H:His98, H:Gly100, and H:Tyr100A) (**Figure 2D**). These residues were found exclusively on CDR loops L3, H3, and H2, with a predominance of heavy chain residues, as expected from visual analysis of the binding cavity. The center of the interaction involved the side-chain of H:Arg95, which was positioned at the bottom of the binding cavity and formed a charged interaction with the carboxyl group of the first sialic acid residue (NeuNAc1) and a hydrogen bond to the Gal saccharide unit. Adjacent to H:Arg95 was L:Tyr92 which hydrogen bonded to both H:Arg95 and the terminal sialic acid (NeuNAc2) residue. Additional hydrogen bonding interactions were seen between the side chain of H:Asn58 to NeuAc2, and the main chain of H:Ala53 and H:Gly54 to NeuNAc1, and the main chain of H:Gly100 to the Glc saccharide unit. Although not directly observed in the docked model, H:His98 and H:Trp52 were in close proximity to the Glc and NeuNAc2 units and most likely contributed aromatic Pi – CH interactions that stabilized the binding. No interactions were found with the GalNac saccharide unit, which was solvent exposed. The orientation of the GalNac did, however, constrain the geometry of the GD2 head group to form a compact fold that defined the binding epitope made up by the adjacent four saccharide units.

### *In silico* Scanning Mutagenesis of 3F8:GD2 Model

*In silico* scanning mutagenesis was performed by taking the 12 residues that directly interacted with GD2 in the docked 3F8:GD2 model (L:Tyr37, L:Lys55, L:Val99, L:Leu102, H:Gly40, H:Tyr31, H:Asn32, H:Asn34, H:Ser56, H:Ser58, H:Gly97, and H:Met98), and analyzing the effect of single point mutations to all possibilities at each site. The models were energy minimized using CHARMm force-fields then analyzed for changes in interaction energies (electrostatic, van der Waals, entropic). The top mutations are shown in **Table 3**. Only 4 mutations were found to increase the interaction energy of the bound complex by more than 1 kcal/mol (**Table 3**). Only one point mutation was predicted to have substantially higher interaction energy (H: Gly54Ile) by a weighted mutation energy of −8 kcal/mol. The majority of this increase in interaction energy was due to an increase in van der Waals contact with the antigen. The effects of double point and triple point mutations involving the 12 interacting residues were also computed, but no additional combination of mutations was found to increase the interaction energy.

**Table 3:** Results of *in silico* scanning mutagenesis of CDR residues that directly interact with docked GD2 antigen. Energies are shown in units of kcal/mol.

Residue	Mutation	VDW Term	Electrostatic Term	EntropyTerm	Weighted MutationEnergy	Effect of Mutation
HC: GLY54	ILE	−18.84	0.21	0.19	−8.23	stabilizing
HC: GLY103	LEU	−5.39	0.23	−0.07	−2.38	stabilizing
HC: GLY103	TRP	−4.44	0.23	−0.01	−1.9	stabilizing
HC: GLY55	THR	−2.96	0.07	−0.1	−1.38	stabilizing

### Analysis of Antigen Binding Site of 3F8 and 3F8-Ile (H:Gly54Ile)

**Figure 3:**
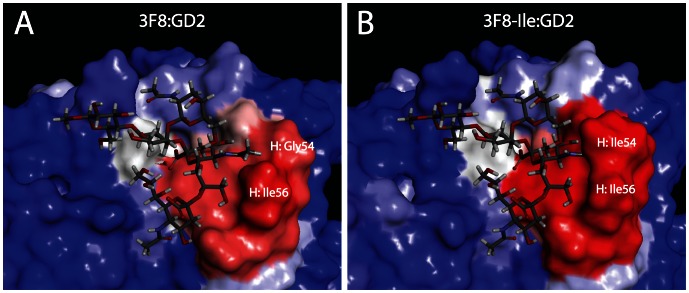
Hydrophobic surface map of MoAb 3F8 and MoAb 3F8-Ile (H:Gly54Ile). Surfaces are rendered using Spatial Aggregation Propensity algorithm [34]. Highly hydrophobic patches are rendered in red, whereas hydrophilic surfaces are rendered in blue. A. MoAb 3F8 contains a hydrophobic patch that centers around H:Ile56. B. MoAb3F8-Ile has a binding pocket that creates additional surface contact with GD2 and contains a larger exposed hydrophobic surface area.

The single point mutation derived from *in* silico scanning mutagenesis simulations (H:Gly54Ile, termed 3F8-Ile) was modeled into the antigen binding site of 3F8 (**Figure 3**). Because of the hydrophobic nature of the H:Gly54Ile mutation, an analysis of the hydrophobicity of the antigen binding site was performed, using the Spatial Aggregation Propensity algorithm (Materials and Methods), which provides a measure of the hydrophobic solvent exposed patches). MoAb 3F8 has a hydrophobic patch at the GD2 binding site that centers around H:Ile56 (**Figure 3A**). H:Ile56 protrudes out of the binding cavity and may help the antibody interact with the membrane surface that surrounds the GD2 head group. Substitution of H:Gly54 to Ile increases the exposed hydrophobic surface area of the antigen binding site and also increases the van der Waals contact with GD2 in the docked model (**Figure 3B**).

### Binding and Tumor Cell Killing Properties of hu3F8 and hu3F8-Ile H:Gly54Ile

**Figure 4:**
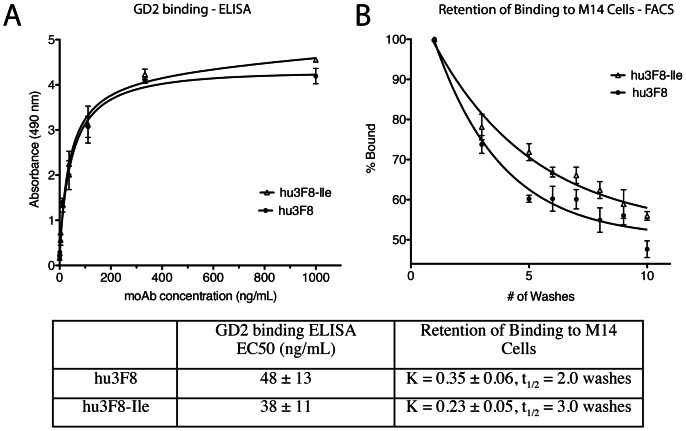
Hu3F8 with H:Gly54Ile has enhanced binding to GD2. A. ELISA assay of hu3F8 and hu3F8-Ile binding to ganglioside GD2 adhered to an ELISA plate. B. Retention of MoAbs hu3F8 and hu3F8-Ile to melanoma M14 cells after successive washes with PBS-EDTA. Decay rate constants and t_1/2_ are shown based on exponential decay curve fits.

To test whether the H:Gly54Ile mutation increases affinity to GD2 and ADCC of tumor cells, the mutation was engineered into the recently described humanized 3F8 (hu3F8) [7]. Hu3F8 is less immunogenic than murine 3F8, retains the structural features of murine 3F8 found in this investigation, and is currently in phase I clinical trials. Hu3F8 and hu3F8 H:Gly54Ile (hu3F8-Ile) were constructed, expressed, purified, and tested for GD2 binding and ADCC. ELISA assays on GD2 showed that hu3F8-Ile had a negligible increase in binding efficiency relative to hu3F8 (EC50 of GD2 binding: hu3F8 48±13 ng/mL, hu3F8-Ile 38±11 ng/mL) (**Figure 4A**). To test the avidity of these antibodies to bind to GD2 in its native environment on the surface of tumor cells, a wash experiment was carried out where antibodies bound to the surface of M14, a GD2(+) melanoma cell line, were subjected to consecutive washing cycles with PBS-EDTA (see Material and Methods). Hu3F8-Ile showed a greater ability to resist being washed off tumor cells (t_1/2_ of hu3F8-Ile = 3 washes, t_1/2_ of hu3F8 = 2 washes) (**Figure 4B**). No other mutation was found to enhance antigen binding.

A control experiment was done to determine whether hu3F8-Ile non-specifically binds to membrane surfaces (**Table 4**). Hu3F8 and hu3F8-Ile were tested for their ability to bind neuroblastoma cells that were GD2-positive (LAN1) or GD2-negative (SK-N-SH and SK-N-AS) by flow cytometry. Both antibodies had similar binding to GD2-positve cells and no binding to GD2-negative cells, demonstrating that hu3F8-Ile retains specificity to GD2 on the membrane surface. The anti-CD20 MoAb Rituximab was used as a negative control.

**Table 4:** Binding to GD2-positive and GD2-negative neuroblastoma cell lines by flow cytometry.

	hu3F8	hu3F8	rituximab
		HC:G54I	
LAN-1 (GD2-postive)	3107±197	2908±173	2±0.2
SK-N-SH (GD2-negative)	7±0.8	3±0.1	2±0.1
SK-N-AS (GD2-negative)	7±0.6	4±0.3	3±0.3

Data is shown as mean florescence intensity±standard deviation. Anti-CD20 MoAb rituximab was used as a negative control.

**Figure 5:**
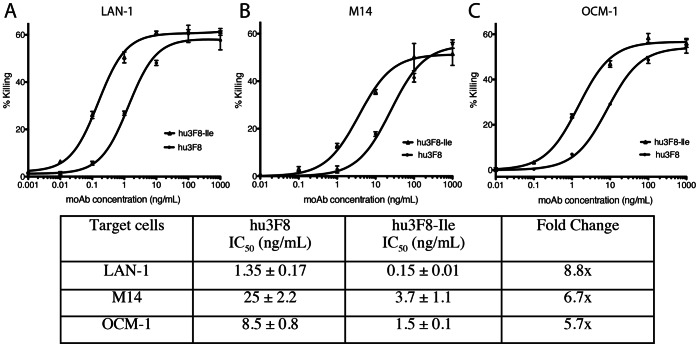
Hu3F8 with H:Gly54Ile has enhanced ADCC. A. ADCC on neuroblastoma LAN-1 cells. B. ADCC on melanoma M14 cells. C. ADCC on melanoma OCM-1 cells. IC50 values shown are based on sigmoidal curve fits.

Hu3F8 and hu3F8-Ile were then assayed for their efficiency in mediating ADCC of neuroblastoma LAN-1 in the presence of natural killer cell line NK-92MI transfected with human CD16 Fc receptor (**Figure 5**). Hu3F8-Ile showed consistently a ∼9-fold increase in cytotoxicity potency compared to hu3F8 (IC50 cell killing: hu3F8 1.35±0.15 ng/mL, hu3F8-Ile 0.15±0.01 ng/mL). A 6–7 fold increase in ADCC potency against melanomas M14 and OCM-1 cells was also observed (IC50 cell killing of M14 cells: hu3F8 25±2.2 ng/mL, hu3F8-Ile 3.7±1.1 ng/mL; IC50 cell killing of OCM-1 cells: hu3F8 8.5±0.8 ng/mL, hu3F8-Ile 1.5±0.1 ng/mL). These increases in ADCC potency for hu3F8-Ile relative to hu3F8 were highly significant (p<0.001).

## Discussion

The clinical efficacy of anti-GD2 MoAbs for the treatment of children with high-risk neuroblastoma has been demonstrated in human clinical trials, and its application to other GD2 positive tumors needs further development. In this study, we report the high-resolution crystal structure of the murine anti-GD2 MoAb 3F8, which has the highest relative affinity among a series of anti-GD2 MoAbs [7]. We have shown that the computational docking algorithm CDOCKER can accurately predict the docked complex of large flexible ganglioside head groups in known co-complex structures found in the protein data bank. Using this method, we created a model of 3F8 bound to the ganglioside head group of GD2 and detailed the key structural elements responsible for antigen recognition. We have additionally applied this method of docking to two other anti-GD2 monoclonal antibodies (ME36.1 and 14G2a) (see **File S1**). Based on the crystal structure of the 3F8 Fab fragment and the comparative analysis, the relatively high affinity of 3F8 to GD2 compared to other antibodies is due to the extensive network of interactions involving main-chain and side-chain hydrogen bonding, two Pi – CH interactions, and an important charged interaction between Arg95 of the H3 loop with the penultimate sialic acid residue of GD2.

To further optimize the potential clinical efficacy of humanized 3F8, we employed high throughput *in silico* scanning mutagenesis on the key interacting residues in the 3F8:GD2 docked model. We identified a single point mutation (H:Gly54Ile) that showed a modest increase in binding affinity in GD2-ELISA assays and an increase in the ability of hu3F8-Ile to retain binding to GD2 on a cell surface. More strikingly, we showed that hu3F8 had a ∼6–9 fold increase in ADCC of GD2-positive tumor cell lines, including neuroblastoma, melanoma, osteosarcoma, and small cell lung cancer. The nature of the H:Gly54Ile mutation increases the exposed hydrophobic surface area at the antigen binding site. Since GD2 is embedded into the membrane surface by a ceramide moiety, the addition of an Ile at the antigen-binding site may potentiate ADCC, by enhancing the ability of MoAb 3F8 to stay bound to the membrane surface, as observed in the cell washing experiments.

In order to attempt the *in silico* scanning mutagenesis strategy, we had to first overcome the obstacle of computationally docking a large flexible carbohydrate ligands, particularly one carrying sialic acid moieties, by verifying that a docking algorithm could successfully predict the bound conformation of carbohydrates similar to GD2. By using test cases from the protein data bank, we utilized CDOCKER to create a docked model of GD2 bound to MoAb 3F8. Although CDOCKER had been shown to be accurate for ligands on the order of 10 rotatable bonds, it had not been previously shown to be accurate for large oligosaccharides, which can easily have more than 30 rotatable bonds. We propose that this method in combination with CHARMm based mutagenesis may be a useful method in engineering enhanced contacts between antibodies and proteins to carbohydrate antigens. There are limitations to this method since enhancement of affinity alone may not result in enhanced ADCC, which is epitope dependent. In our study, the epitope was located at the putative membrane interaction site, which may have potentiated the enhanced tumor cell killing.

Carbohydrate antigens play an important role in several biological pathways. The development of antibodies to target carbohydrates is important for investigating bacteria, tumors, blood groups, cell-cell adhesion interactions; viral, hormone, and toxin receptors; and the glycosylation of recombinant proteins [28]. Because the immune response to saccharides is T-cell independent, antibodies generated towards carbohydrate antigens are often produced as low affinity IgM antibodies [28]. In order to generate higher affinity antibodies for therapeutic application as in the case for cancer immunotherapy, affinity maturation techniques often need to be employed to enhance therapeutic effect. Traditional methods of antibody affinity maturation such as yeast/phage/ribosomal display rely on error-prone PCR that may not provide the full range of diversity at each of the amino acids in the CDR of the antibody. In this investigation we show that *in silico* scanning mutagenesis could be employed even if a high-resolution co-complex structure is not available. We additionally demonstrate that a modest increase in affinity can enhance the functional properties of MoAb 3F8 for therapeutic targeting to the tumor antigen GD2. Although an enhancement of ADCC is expected to translate into improved efficacy, this will have to be proven in a future clinical trial in patients. The use of these *in silico* techniques may provide a valuable addition to traditional experimental methods in developing the next generation of MoAb for the diagnosis or the treatment of not just cancer, but other human disorders where carbohydrate epitopes are druggable targets.

## Materials and Methods

### Antibody Purification of Murine 3F8 and Fab Fragment Preparation

Murine anti-GD2 MoAb 3F8 (IgG3) was purified from concentrated hybridoma supernatant, as previously described [29]. Fab fragments of m3F8 were generated by papain digestion using a standard Fab preparation kit (Pierce Biotechnology, Rockford, IL).

### Crystallization and Data Collection

The purified 3F8 Fab fragment was concentrated to 12 mg/ml in 20 mM HEPES pH 6.5 and was crystallized in a hanging drop by vapor diffusion at 16°C against a reservoir containing Hampton Index reagent D7 containing 0.1M BIS-TRIS, pH 6.5, 25% PEG 3350 (Hampton Research, Aliso Viejo, CA). The droplet was formed by mixing 1 µl of protein solution and 1 µl of reservoir solution. The crystals were protected by cryoprotectant containing 25% glycerol, 0.1M BIS-TRIS, pH 6.5, 25% PEG 3350. Data was collected at the Argonne Advanced Photon Source beamline 24IDC. The crystals belonged to the space group C2 and diffracted to 1.65 Å resolution.

### Structure Determination and Refinement

The Fab structure was solved by molecular replacement with search model PDB entry 2AJU using Phaser (CCP4 suite) [30]. The best molecular replacement model was refined using Refmac5 [31], manual fitting was performed with O [32], adding solvent with Arp–Warp [33]. The final model contained two polypeptide chains of m3F8 Fab and 585 solvent molecules. Data refinement statistics are shown in Table 1. The final model was deposited in the Protein Data Bank (access code 3VFG).

### Molecular Docking Simulations and *in silico* Mutagenesis

GLIDE docking was performed using Schrodinger Suite 2009 platform (Schrödinger, New York, NY). OPLS force fields were used to parameterize the proteins and ligands. Top ligand poses were clustered within a root-mean-square deviation of 2.0 Å and scored by GlideScore. CDOCKER docking and interaction energy measurements were performed using Discovery Studio 3.0 (Accelrys, San Diego, CA). CHARMm force fields were used to parameterize the proteins and ligands. Top ligand poses were clustered within a root-mean-square deviation of 2.0 Å and scored by CDOCKER Interaction Energy. For all docking studies involving GD2, the ceramide tail was replaced by a methyl group (Figure 1). Docking simulations were done under rigid-body conditions where ligand conformations were docked onto proteins/antibodies with rigid side chains. Final docked complexes were energy minimized with CHARMm using Smart Minimizer algorithm on Discovery Studio 3.0 (Accelrys, San Diego, CA). *In silico* mutagenesis was done by calculating the free energy of binding of the docked antibody:antigen model using CHARMm force fields and the Calculate Mutation Energy protocol on Discovery Studio 3.0 (Accelrys, San Diego, CA).

### Image Rendering

Molecular structure images were rendered with Pymol (Schrödinger, New York, NY) for docking studies, or with Discovery Studio 3.0 (Accelrys, San Diego, CA) for electrostatic potential surfaces.

### Modeling of Exposed Hydrophobic Surface Area

The antigen binding site of MoAb 3F8 and MoAb 3F8 H:Gly54Ile was modeled on Discovery Studio 3.0 (Accelrys, San Diego, CA). Exposed hydrophobic surfaces were rendered using Spatial Aggregation Propensity algorithm developed by Chennamsetty et al. [34], where patches of effective dynamically exposed hydrophobicity on a protein surface is quantitated and colored in red.

### Cell Culture

Human neuroblastoma cell line LAN-1 [35] was provided by Dr. Robert Seeger (Children’s Hospital of Los Angeles). Melanoma cell lines M14 [36] and OCM-1 [37] from Dr. David Cobrinik (Children’s Hospital of Los Angeles). Neuroblastoma cell lines SK-N-SH and SK-N-AS were obtained from ATCC (Manassas, VA). All cell lines were grown in F10 [RPMI 1640 medium supplemented with 10% fetal bovine serum (Hyclone, South Logan, UT), 2 mM glutamine, 100 U/ml penicillin, and 100 µg/ml streptomycin at 37°C in a 5% CO_2_ incubator.

### Construction of the hu3F8 and Variants

Humanized 3F8 genes were synthesized for CHO cells (Blue Heron Biotechnology or Genscript) as previously described [7]. Using the bluescript vector (Eureka, CA), these heavy and light chain genes of hu3F8 were transfected into DG44 cells and selected with G418 (InVitrogen, CA).

### Purification of Antibodies

Hu3F8 and chimeric 3F8 producer lines were cultured in Opticho serum free medium (InVitrogen) and the mature supernatant harvested as previously described [7]. Protein A affinity column was pre-equilibrated with 25 mM sodium citrate buffer with 0.15 M NaCl, pH 8.2. Bound hu3F8 was eluted with 0.1 M citric acid/sodium citrate buffer, pH 3.9 and alkalinized (1∶10 v/v ratio) in 25 mM sodium citrate, pH 8.5. It was passed through a Sartobind-Q membrane and concentrated to 5–10 mg/ml in 25 mM sodium citrate, 0.15 M NaCl, pH 8.2.

### Quantitation of GD2 Binding by ELISA and Flow Cytometry

ELISA was performed as previously described [7]. Microtiter plates were coated with GD2 at 20 ng per well. 150 µl per well of 0.5% BSA in PBS (diluent) was added to each plate for at least 30 min at ambient temperature to block excess binding sites. 100 µl of standard and samples (diluted 2-fold) were added to each well and incubated for 2.5 h at 37°C. After washing the plates with PBS, 100 µL of goat anti human-IgG (H+L) (Jackson Research Laboratory) diluted at 1∶3500 in diluent was added to each well and incubated for 1 h at 4°C. ELISA color reaction was developed with chromogen OPD (Sigma) with the substrate hydrogen peroxide for 30 min at ambient temperature in the dark. The reaction was stopped with 5N H_2_SO_4_ and the optical density (OD) read with ELISA plate reader MRX (Dynex) at 490 nm.

To measure the retention of binding of MoAbs to antigen containing cells, antibodies were incubated with melanoma M14 cells and successively washed off. Cells were initially collected at 1×10^6^ cells per round bottom tube, centrifuged and rinsed with PBS, and resuspended in 100 µL PBS per assay tube. Cells were incubated with MoAbs hu3F8 or hu3F8-Ile ((1 µg MoAb/1×10^6^ cells) for 30 minutes at 4°C. Cells then underwent successive rounds of washing using 5 ml PBS with 3 mM EDTA, followed by pelleting, discarding of supernatant and resuspension. With each successive wash, samples were incubated with R-Phycoerythrin (R-PE) conjugated anti-human IgG, Fcγ fragment specific secondary antibody (Jackson ImmunoResearch) for 30 minutes at 4°C in the dark, washed, and then analyzed by flow cytometry using a BD FACS Calibur instrument. Samples were prepared in triplicate. Flow cytometry was used to measure antibody binding to GD2-positive LAN-1 cells and GD2-negative SK-N-SH and SK-N-AS cells using the same R-Phycoerythrin (R-PE) conjugated anti-human IgG, Fcγ fragment specific secondary antibody (Jackson ImmunoResearch). Rituximab (Genentech, South San Francisco, CA) was used as a control antibody.

### Antibody-dependent Cell-mediated Cytotoxicity (ADCC) by ^51^chromium Release

ADCC assays were performed using NK-92MI cells stably transfected with the human CD16 Fc receptor as previously described [7]. LAN1-1, M14, OCM-1, U2OS, CRL1427, NCI-H345 target cells were detached with 2 mM EDTA in Ca2+ Mg2+ free PBS and washed in F10, before radiolabeling with ^51^Cr for ADCC assays.

### Statistical Analyses

Curve fitting and statistical analyses were performed using GraphPad Prism 5.0. Student’s T-test was used for calculations of significance.

## Supporting Information

File S1 **Supporting information.** Figure S1. Docking studies of ME36.1 and 14G2a with GD2 pentasaccharide. A. Docked model of GD2 pentasaccharide with ME36.1 Fab crystal structure. B. Docked model of GD2-pentasaccharide with 14G2a homology model. Figure S2. Electrostatic potential surfaces of 3F8, ME36.1, and 14G2a. A. Electrostatic potential surfaces of CDR regions. B. Electrostatic potential surfaces with docked GD2 pentasaccharide. Relative orientations of ceramide moieties are indicated.
(DOCX)
