# Supplementary material for: In silico Driven Redesign of a Clinically Relevant Antibody for the Treatment of GD2 Positive Tumors
Source: PLoS One. 2013 May 16;8(5):e63359. doi: 10.1371/journal.pone.0063359 (PMC3656052; doi:10.1371/journal.pone.0063359)
Supplement: File S1 — Supporting information. Figure S1. Docking studies of ME36.1 and 14G2a with GD2 pentasaccharide. A. Docked model of GD2 pentasaccharide with ME36.1 Fab crystal structure. B. Docked model of GD2-pentasaccharide with 14G2a homology model. Figure S2. Electrostatic potential surfaces of 3F8, ME36.1, and 14G2a. A. Electrostatic potential surfaces of CDR regions. B. Electrostatic potential surfaces with docked GD2 pentasaccharide. Relative orientations of ceramide moieties are indicated. (DOCX) [file pone.0063359.s001.docx]

**Supporting Information**

*In silico* driven redesign of a clinically relevant antibody for the treatment of GD2 positive tumors

Mahiuddin Ahmed^a^, Yehuda Goldgur^b^, Jian Hu^a^, Hong-Fen Guo^a^ and Nai-Kong V. Cheung^a,1^

**Affiliations:**

^a^Department of Pediatrics, Memorial Sloan-Kettering Cancer Center, 1275 York Avenue, New York, NY 10065, USA.

^b^Structural Biology Program, Memorial Sloan-Kettering Cancer Center, 1275 York Avenue, New York, NY 10065, USA.

^1^Correspondence to: Nai-Kong V. Cheung, MD PhD, Department of Pediatrics, Memorial Sloan-Kettering Cancer Center, 1275 York Avenue, New York, NY 10065, USA. Tel No. 646-888-2313; Fax No. 646-422-0452; E-mail: [cheungn@mskcc.org](mailto:cheungn@mskcc.org)

**
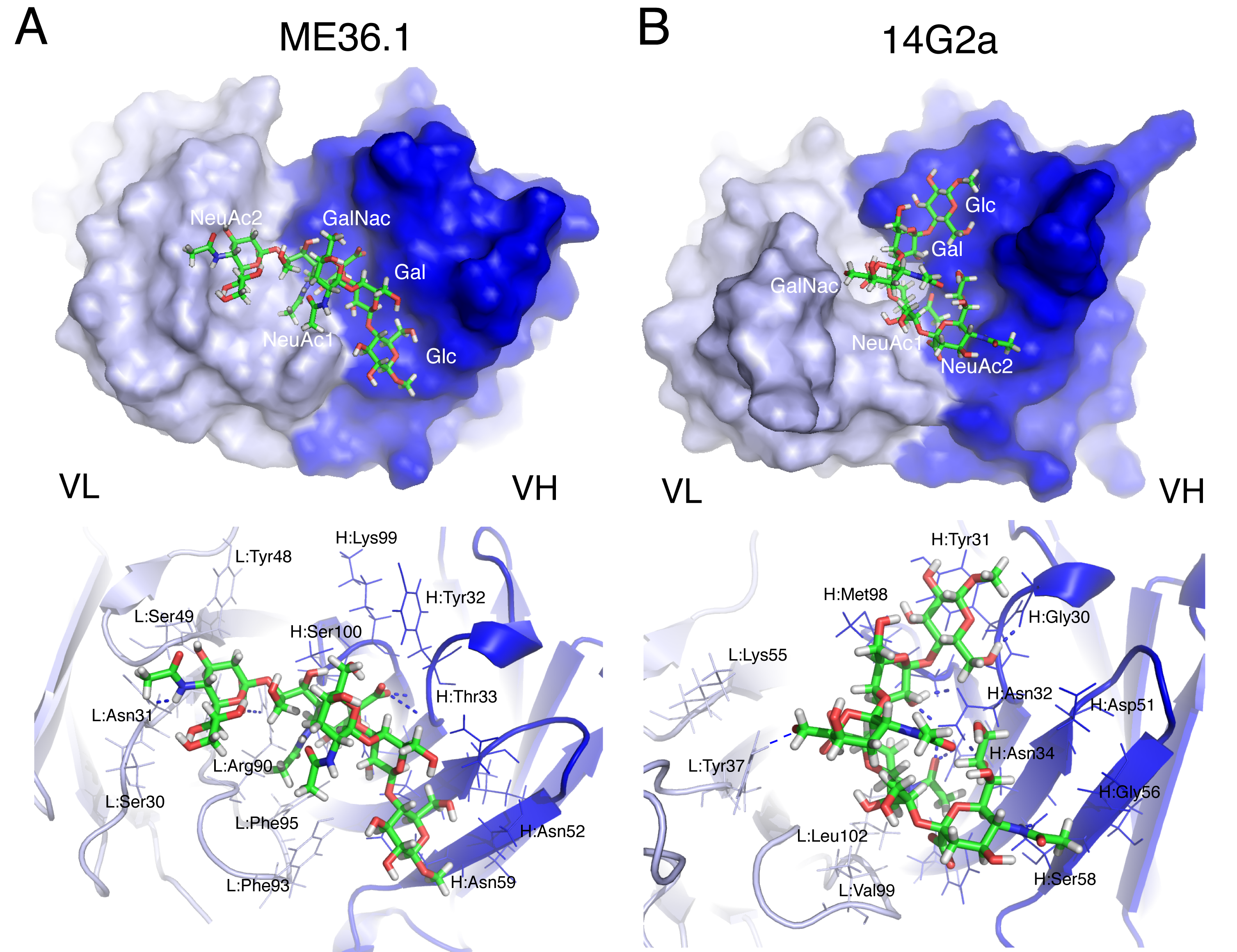
**

**Figure S1. Docking studies of ME36.1 and 14G2a with GD2 pentasaccharide.** A. Docked model of GD2 pentasaccharide with ME36.1 Fab crystal structure. B. Docked model of GD2-pentasaccharide with 14G2a homology model.

**
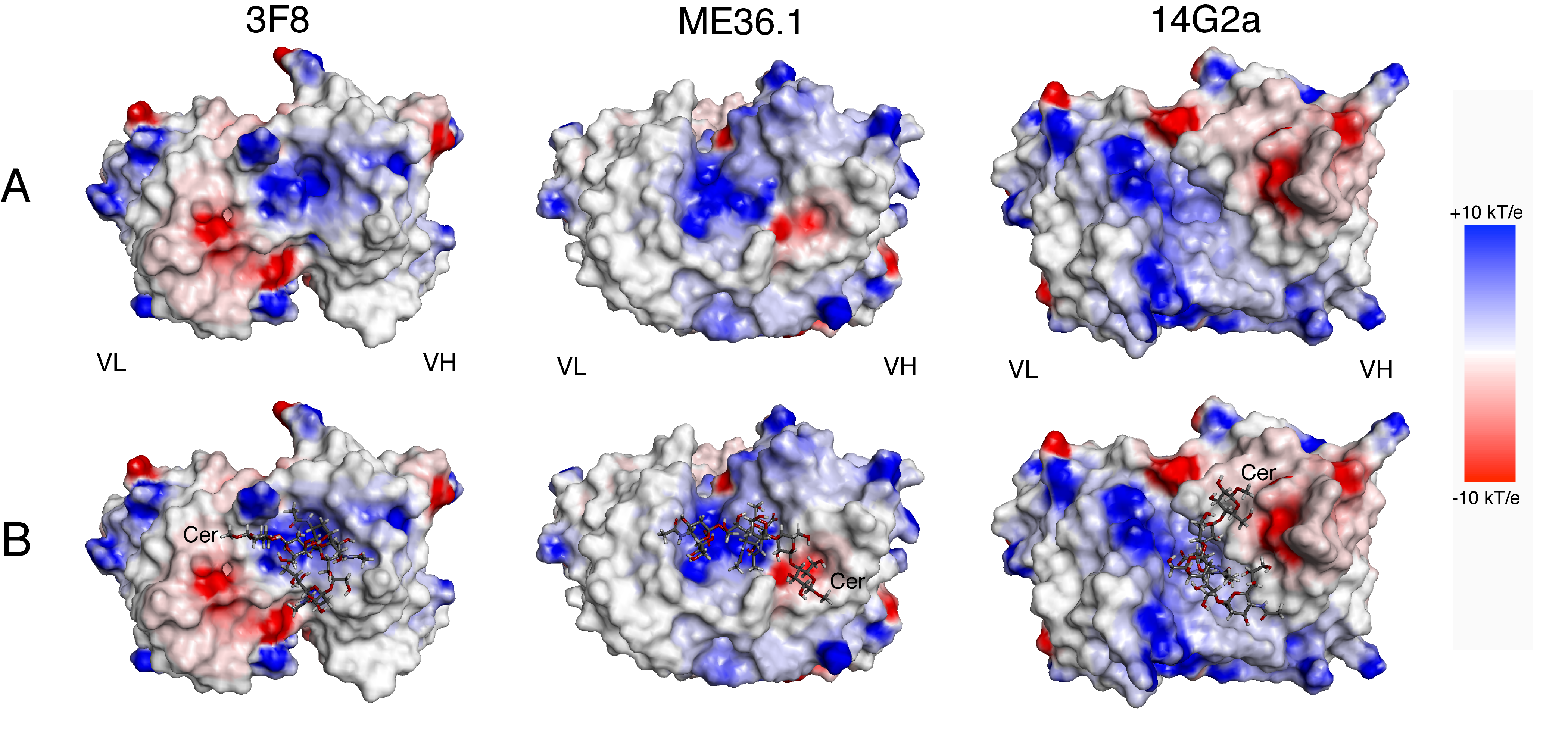
**

**Figure S2. Electrostatic potential surfaces of 3F8, ME36.1, and 14G2a.** A. Electrostatic potential surfaces of CDR regions. B. Electrostatic potential surfaces with docked GD2 pentasaccharide. Relative orientations of ceramide moieties are indicated.

**Supporting Information Results**

**Docking studies of GD2 to MoAbs ME36.1 and 14G2a**

To understand the molecular basis for the dissimilar binding affinities of anti-GD2 MoAbs, docking studies using CDOCKER were applied to the crystal structure of ME36.1 (pdb 1PSK) and a homology model of 14G2a. The homology model of 14G2a was made using the Rosetta Antibody Modeling server, followed by loop refinement and CHARMm energy minimization (see Methods). The docked complexes of GD2 with ME36.1 and 14G2a are presented in **Figure S1**.

ME36.1 had a more elongated binding pocket than what was found in 3F8. Similar to 3F8, the docked model of ME36.1 with GD2 showed that binding was determined by an extensive hydrogen bonding and electrostatic interaction involving several polar amino acids and a central charged Arg (L: Arg90) that anchored the complex. There were a total of 13 amino acids that directly interacted with GD2 (L:Asn31, L:His33, L:Ser49, L:Arg90, L:Tyr93, H:Tyr32, H:Thr33, H:His35, H:Asp50, H:Asn52, H:Gly57, H:Asn59, and H:Ser100). In the GD2 head group, NeuAc1 was held into the binding pocket with L:Arg90 charged interaction and hydrogen bonding with L:Asn31. Additional side-chain hydrogen bonding was observed between H:Thr33 and H:His35 with NeuAc1. Main-chain hydrogen bonding was observed between the amine group of H:Tyr32 and the carboxyl group of NeuAc1. Although not directly observed, several of the other polar residues were within hydrogen bonding distances that could occur dynamically, including L:Ser30, L:Ser49, L:Tyr93, H:Asn59, H:Asn52, and H:Ser100. As in the docked complex of 3F8 with GD2, no contacts were observed with the GalNac unit, which pointed out into the solvent. The binding epitope would therefore not contain GalNac; this might explain how ME36.1 would bind with modest affinity to GD3, which is identical to GD2 minus GalNac. The binding pocket of ME36.1 was also more elongated than the compact pocket found in 3F8. GD3, without the constraints of a branched GalNac unit, would have a more elongated structure in its low energy state and could occupy the same epitope space as predicted in the docked model.

The docked complex of 14G2a with GD2 showed a different binding mode from that of 3F8 or ME36.1. The binding interaction involved 12 residues (L:Tyr37, L:Lys55, L:Val99, L:Leu102, H:Gly40, H:Tyr31, H:Asn32, H:Asn34, H:Ser56, H:Ser58, H:Gly97, and H:Met98). Unlike 3F8 and ME36.1, no central Arg was found in the binding pocket. There were, however, several polar amino acids that could form hydrogen bond as well as electrostatic interactions with the GD2 antigen. At the center of the binding pocket were two Asn residues (H:Asn32 and H:Asn34) which anchored the antigen to the binding pocket and formed hydrogen bonds with the NeuAc1 saccharide unit. Additional hydrogen bonds were formed between the side-chain of H:Syr58 and NeuAc2, as well as the main-chain H:Gly39 and H:Asn32 with Glc. The GalNac in this model also remained solvent exposed, but did form a hydrogen-bond with L:Tyr37. Although not directly observed in this model, a charged interaction could form between L:Lys55 and the carboxyl group of NeuAc1, which were 6 Å apart.

**Electrostatic analysis of 3F8, ME36.1 and 14G2a**

Electrostatic potential maps were generated of 3F8, ME36.1 and 14G2a using DelPhi (**Figure S2**). The binding pockets of 3F8 and Me36.1 were characterized by a dense area of positive charge formed by H:Arg95 and His:98 in 3F8, and by L:Arg90 and L:His33 in ME36.1. The docked models showed that these regions were in the center of the respective binding pockets and assisted in neutralizing the -2 formal charge of the sialic acid units in the GD2 head group. In the model of 14G2, a smaller area of positive charge was seen (L:His39, L:His54, and L:Lys55). This region was in proximity to the docked sialic acid groups of GD2 and may have contributed to stabilizing the docked complex.

**Supporting Information Discussion**

We implemented CDOCKER in combination with CHARMm force field based minimizations to create docked models of GD2 bound to 3F8, and two other anti-GD2 antibodies ME36.1 and 14G2a.

Comparison of the three docked complexes of anti-GD2 MoAbs highlighted the key electrostatic, aromatic, and van der Waaals interactions that determined the relative specificities and affinities of 3F8, ME36.1, and 14G2a. Surface plasmon resonance measurements have previously shown that 3F8 and ME36.1 have considerably higher affinities for GD2 than 14G2a [[1](#_ENREF_1)]. In this analysis, the key structural features were identified that were common to 3F8 and ME36.1, including the presence of central Arg and His side chains that anchored the negatively charged GD2 head group, and the predominance of polar residues that created extensive hydrogen bonding networks with the large flexible oligosaccharide antigen. 14G2a had a similar positively charged surface formed by Lys and His residues that were positioned away from the center of the binding pocket that also helped to neutralize the negatively charged sialic acid groups of GD2. The higher positive charge of Arg (pKa ~12.5) versus Lys (pKa ~10.5) might have contributed to the higher affinity of 3F8 and ME36.1 relative to 14G2a.

In terms of specificity, 3F8 and 14G2a have a higher specificity for GD2 binding than ME36.1 which had modest cross-reactive binding to GD3 [[2](#_ENREF_2)]. Due to the presence of a branched GalNac saccharide unit, the docked complexes showed that GD2 bound to 3F8 or 14G2a had a compact fold that mimicked the low energy state of unbound GD2,. ME36.1 had a more elongated binding pocket that could encompass the low energy state of the GD3 head group which has an unbranched tetrasaccharide. The higher affinity of 3F8 is derived from the involvement of the 12 interacting amino acids residues in the CDR forming a charged interaction with H:Arg95, two Pi-CH interactions with H:Trp52 and H:His98, and the extensive hydrogen bonding plus electrostatic interactions with the predominantly polar side-chains in the CDR. This analysis also revealed key structural features that stabilized the variable-heavy chain and variable-light chain interface, which allows for the proper orientation of the CDR loops.

The generation of humanized MoAbs from their murine parental form is a key impetus in the development of therapeutically effective anti-tumor treatment regimens. The majority of patients receiving murine anti-GD2 antibodies develop human anti-mouse antibodies (HAMA) that could delay treatment, and limit the total dose or number of cycles administered. Humanization of ME36.1 was previously reported as difficult [[2](#_ENREF_2)]. Chimerization and humanization of 14G2a were successful, leading to phase III of chimeric 14.18 (a class variant of 14G2a) [[3](#_ENREF_3)] and various versions of the humanized form including hu14.18-IL2[[4](#_ENREF_4)]and hu14.18-K322A [[3](#_ENREF_3)]. Humanization of 3F8 (hu3F8) was recently done by CDR grafting methods and homology modeling [[1](#_ENREF_1)], and is currently in phase I trials for the treatment of neuroblastoma and sarcomas (NCT01419834, clinicaltrials.gov). Hu3F8 retained the overall structure of murine 3F8 crystal structure described in this report.

**Supporting Information Methods**

**Homology modeling**

Homology modeling of the variable region of antibody 14G2a was done by means of ROSETTA antibody server [[5](#_ENREF_5)] using the full protocol with VL-VH refinement, consisting of detecting templates, grafting CDRs, and modeling the CDR H3 with simultaneous minimization of CDR backbone conformations and relative orientation of the light (VL) & heavy (VH) chains. Loop refinement was performed using Discovery Studio 3.0 (Accelrys, San Diego, CA). Templates from the PDB used for framework and loops were as follows: heavy chain framework 1KB5, light chain framework 1JGU, H1 loop 1KB5, H2 loop 1KB5, H3 loop 1H8N, L1 loop 1QKZ, L2 loop 3IFO, L3 loop 1JGU.

**Electrostatic surface mapping**

DelPhi [[6](#_ENREF_6)] was used to calculate the electrostatic surface maps for MoAbs 3F8, ME36.1 and 14G2a. Surfaces were plotted as a function of energy in kT/e.

**REFERENCES**

1. Cheung NK, Guo H, Hu J, Tassev DV, Cheung IY (2012) Humanizing murine IgG3 anti-GD2 antibody m3F8 substantially improves antibody-dependent cell-mediated cytotoxicity while retaining targeting in vivo. Oncoimmunology 1: 477-486.

2. Pichla SL, Murali R, Burnett RM (1997) The crystal structure of a Fab fragment to the melanoma-associated GD2 ganglioside. J Struct Biol 119: 6-16.

3. Sorkin LS, Otto M, Baldwin WM, Vail E, Gillies SD, et al. (2010) Anti-GD(2) with an FC point mutation reduces complement fixation and decreases antibody-induced allodynia. Pain 149: 135-142.

4. Delgado DC, Hank JA, Kolesar J, Lorentzen D, Gan J, et al. (2010) Genotypes of NK Cell KIR Receptors, Their Ligands, and Fc gamma Receptors in the Response of Neuroblastoma Patients to Hu14.18-IL2 Immunotherapy. Cancer Res 70: 9554-9561.

5. Sircar A, Kim ET, Gray JJ (2009) RosettaAntibody: antibody variable region homology modeling server. Nucleic Acids Research 37: W474-W479.

6. Rocchia W, Sridharan S, Nicholls A, Alexov E, Chiabrera A, et al. (2002) Rapid grid-based construction of the molecular surface and the use of induced surface charge to calculate reaction field energies: Applications to the molecular systems and geometric objects. J Comput Chem 23: 128-137.
